# The Presence of Anti-Angiotensin II Type-1 Receptor Antibodies Adversely Affect Kidney Graft Outcomes

**DOI:** 10.3390/ijerph14050500

**Published:** 2017-05-09

**Authors:** Jian Zhang, Mingxu Wang, Jun Liang, Ming Zhang, Xiao-Hong Liu, Le Ma

**Affiliations:** 1 The First Affiliated Hospital, Xi’an Jiaotong University College of Medicine, 277 Yanta West Road, Xi’an 710061, China; www5589759@stu.xjtu.edu.cn; 2 Key Laboratory of Shaanxi Province for Craniofacial Precision Medicine Research, College of Stomatology, Xi’an Jiaotong University, Xi’an 710004, China; 3 School of Public Health, Xi’an Jiaotong University Health Science Center, 76 Yanta West Road, Xi’an 710061, China; wangmx601@xjtu.edu.cn (M.W.); liangjunl@stu.xjtu.edu.cn (J.L.); 4 Xi’an Honghui Hospital, 555 Friendship Road, Xi’an 710054, China; 5 Key Laboratory of Environment and Genes Related to Diseases (Xi’an Jiaotong University), Ministry of Education of China, Xi’an 710061, China

**Keywords:** angiotensin II type 1 receptor antibody, angiotensin II receptor, kidney transplantation, acute rejection, meta-analysis

## Abstract

The aim of this study was to determine whether anti-angiotensin type 1 receptor antibodies (AT1R-Abs) are related to acute rejection (AR) and kidney graft failure in renal transplantation. We searched electronic databases including MEDLINE, EMBASE, and the ISI Web of Science databases for all studies on the association between anti-angiotensin type 1 receptor antibodies and kidney allograft outcomes updated to November 2016. Reference lists from included articles were also reviewed. The pooled relative risks (RRs) with 95% confidence intervals (CIs) were extracted or calculated using a random-effects model. The potential sources of heterogeneity and publication bias were estimated. Nine studies enrolling 1771 subjects were retrieved in the meta-analysis. AT1R-Abs showed significant associations with increased risk of AR (RR = 1.66; 95% CI, 1.23–2.09). In addition, a significant relationship was found between AT1R-Abs and kidney graft failure compared with AR (RR = 3.02; 95% CI, 1.77–4.26). The results were essentially consistent among subgroups stratified by participant characteristics. These results demonstrated that the AT1R-Abs were associated with an elevated risk of kidney allograft outcomes, especially with kidney graft failure. Large-scale studies are still required to further verify these findings.

## 1. Introduction

Kidney transplantation following end-stage renal disease has proved to be the optimal treatment providing notable improvement in patient “quality of life“ [1]. Acute vascular rejection after kidney transplantation is always the most important challenge for sustaining continued long-term function of the allograft [2]. Advances in human leukocyte antigen (HLA) tissue typing and HLA-antibody detection have remarkably improved antibody-mediated rejection (AMR) prediction and recognition [3]. However, acute vascular rejection that is refractory to therapy still occurs in HLA-identical sibling transplants. A variety of non-HLA antibodies have been identified in serum obtained before transplantation from patients in whom refractory rejection developed after they received kidney transplants from HLA-identical siblings [4]. Elucidation of the association between non-HLA antigens and vascular rejection might provide new insight into potential mechanisms, and facilitate the development of specific therapies.

As special non-HLA antibodies, anti-angiotensin type 1 receptor antibodies (AT1R-Abs) are proposed to set up an alternative mechanism for renal graft injury and acute rejection [5]. Anti-angiotensin II type 1 receptor (AT1R) belongs to the type A family of G protein-coupled receptor (GPCR) and is responsible for most angiotensin II-mediated physiological activities, including blood pressure regulation and fluid and electrolyte balance [6]. AT1R-Abs were characterized as immunoglobulin G1 (IgG1) and IgG3 subclass antibodies, which recognize conformational antigens contained in the second extracellular loop of the AT1R. The binding of antibodies to AT1R appears to be capable of inducing excessive activation of signal transduction in vessel endothelial and smooth muscle cells, which was associated with vascular inflammatory damage [7]. Previous studies have shown that AT1R-Abs were directly involved in the vascular disease pathology of hypertension, preeclampsia, and systemic sclerosis [8,9,10], which may share a similar inflammatory mechanism with acute rejection (AR) in transplant recipients with AT1R-Abs. Moreover, AR after kidney allograft transplantation frequently progresses to persistent chronic rejection and dysfunction of kidney allograft, and ultimately results in kidney graft failure [11], indicating that AT1R-Abs may also influence the long-term outcomes of kidney allograft transplantation. Currently, several studies have shown that AT1R-Abs might be associated with an increased risk of acute rejection and kidney graft failure [12,13,14]; however, the results are inconsistent and inconclusive [15,16].

Therefore, we conducted a meta-analysis of the evidence to evaluate the relationship between AT1R-Abs and the risk of AR in renal transplantation. Furthermore, we also examined the impact of AT1R-Abs on long-term kidney graft outcomes.

## 2. Materials and Methods 

### 2.1. Search Strategy

We searched electronic databases including MEDLINE, EMBASE, and the ISI Web of Science databases for all studies on the association between AT1R-Abs and kidney allograft outcomes updated in November 2016, using the search terms: (“angiotensin II type-1 receptor antibody” or “AT1R-Ab” or “AT1R antibody” or “anti-AT1R antibodies” or “AT1Rab”) and (“renal” or “kidney”) and (“transplantation” or “transplant”). No language restriction was imposed on searching and study inclusion. We also checked the reference lists of retrieved articles and relevant reviews to find other potential articles. We attempted to contact the authors and experts of ongoing research when more detailed information was necessary.

### 2.2. Study Selection

To identify all eligible studies, we used a two-step selection strategy. In the first step, we performed an initial review of all identified abstracts and titles to exclude any clearly unrelated articles. Then, the full texts of the remaining studies were further examined for their suitability for the present meta-analysis. The selected references of the full-text articles were checked using the same criteria. Studies included in the present meta-analysis have to meet the following criteria: (1) assessed the association between AT1R-Abs and kidney allograft outcomes (AR or kidney graft failure) among adult renal transplants; (2) used cohort, case-control, or cross-sectional design; (3) provided adjusted relative risk (RR) or odds ratios (OR) with the corresponding 95% confidence interval (CI) or sufficient data to estimate them. When multiple publications reported on the same or overlapping data, we only selected the most updated data. Two reviewers independently screened and assessed publications for potential inclusion in the analysis according to the same criteria. Discrepancies were resolved by a third author (Le Ma).

### 2.3. Data Extraction and Quality Assessment

The following information was extracted from each article: first author; year of publication; study design; research center; sex distribution; average age; first transplant rate; patients with living donors; detection of AT1R-Abs; follow-up period; diagnosis of AR; classification of AR; induction and maintenance regimens; controlled variable. The RRs (or ORs) with 95% CI in the studies were also extracted. If a study provided several risk estimates, we extracted the estimate that reflected the greatest degree of adjustment. The methodological quality of the eligible studies was assessed using Newcastle–Ottawa Quality Scale (NOS) [17]. The quality of each study was assessed and awarded stars for indicators of quality, including three aspects: subject selection (0–4 scores), comparability (0–2 scores) and exposure (0–3 scores). Total scores ranged from 0 (worst) to 9 (best). Studies with a score of 5–9 were considered to be of high quality and studies with a score of 0–4 were considered to be of low quality. Two authors independently extracted data from each study included in the present meta-analysis using standardized data extraction forms. Discrepancies between two authors were resolved by discussion with a third investigator (Le Ma).

### 2.4. Statistical Analysis

RRs with corresponding 95% CI were used to assess the strength of the association between AT1R antibodies and kidney allograft outcomes. Because the absolute risk of AR or kidney graft failure in renal transplantation was low, ORs and hazard ratios (HRs) could also be considered an approximation of relative risk. Summary RRs were calculated using a random effects model. We evaluated heterogeneity between studies with the I^2^ statistic (I^2^ > 50% indicated evidence of heterogeneity) [18]. We explored potential sources of heterogeneity with stratified analyses. Subgroup analysis was conducted by mean age, study design, living donors rate, first transplant rate, adjustment, country of origin. We also performed sensitivity analyses by removing each study one at a time to confirm the stability of the results. Potential publication bias was assessed using Begger funnel plots and the Egger linear regression test (*p* < 0.05 was considered statistically significant) [19,20]. All statistical analyses were performed using the software Stata version 11.0 (StataCorp, College Station, TX, USA).

## 3. Results

**Figure 1:**
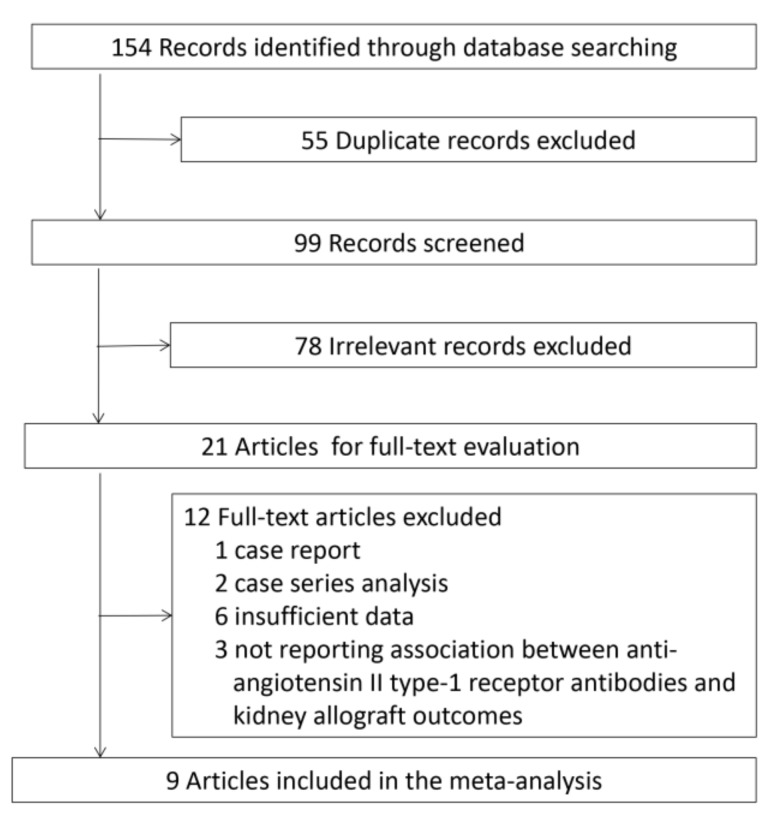
Flowchart showing the study selection procedure.

A systematic search yielded 154 records in total. After excluding duplicates, the titles and abstracts from the remaining 99 records were screened. Of these, 21 articles were selected for full text review, and nine articles were ultimately retained in our meta-analysis (see Figure 1) [12,13,14,15,16,21,22,23,24].

### 3.1. Characteristics of the Studies

The characteristics of the included studies are presented in Table 1. Of the nine studies, five were conducted in America, two in Europe, one in Asia and one in Australia. Six studies were cohort studies, and three were case-control studies. The number of subjects ranged from 70 to 599. In six studies, more than 90% of subjects were receiving a first kidney transplant. The average age of subjects ranged from 27.7 years to 51.3 years. AR was biopsy-proven in all studies, except one study which reported that a 25% increase in serum creatinine was diagnosed as acute rejection. Seven studies employed an induction regimen strategy including anti-thymocyte globulin (ATG) and anti-human interleukin-2 receptor (anti-IL2R) antibody, whereas two studies did not report the induction regimen used. Five studies included reported a triple immunosuppressive therapy with tacrolimus/cyclosporine A (TAC/CsA), mycophenolate mofetil (MMF), and steroids. Two studies reported that TAC/MMF were used; two studies did not report the immunosuppressive therapy employed. All studies included were classified as high quality.

### 3.2. The Presence of AT1R-Abs and AR Risk

**Figure 2:**
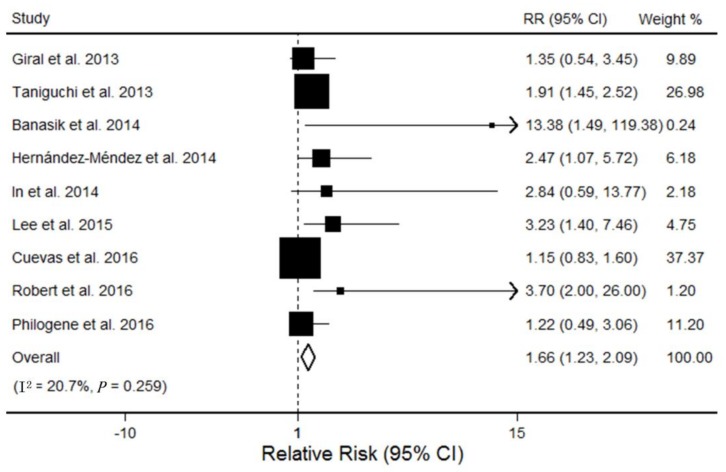
Forest plot on the association between AT1R-Abs and AR. For each study, the estimation of RR and its 95% confidence interval (CI) are plotted with a box and a horizontal line. The pooled odds ratio is represented by a diamond. The area of the gray squares reflects the weight of the study in the meta-analysis.

**Figure 3:**
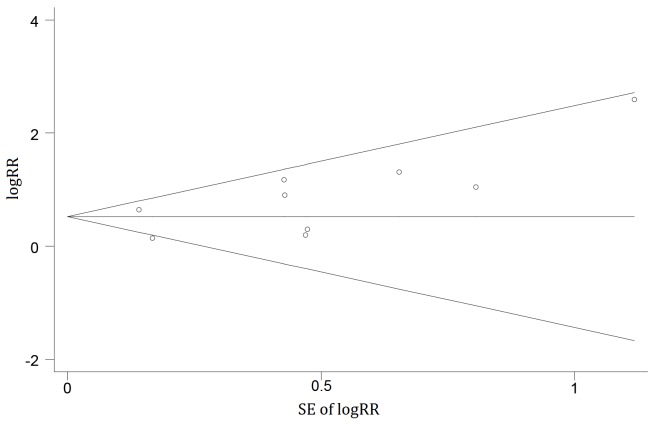
Funnel plots with 95% CI for AT1R-Abs and acute rejection (AR). RR, relative risk; SE, standard error.

Nine studies with a total of 1771 participants reported the relationship between AT1R-Abs and AR. Five included studies show an association between AT1R-Abs and a significantly increased risk of AR, whereas other studies show no relationship between them. Across the nine studies included, patients with AT1R-Abs were associated with a higher RR of developing AR compared with patients without AT1R-Abs (pooled RR, 1.66; 95% CI, 1.23–2.09), using the random effects model (see Figure 2). No evidence of heterogeneity was detected across these studies (I^2^ = 20.7%; *p* = 0.26). Stratified analysis found that none of the participant characteristics substantially altered the shape of the association (see Table 2). Sensitivity analyses indicated that the pooled RRs were not influenced excessively by any single study. The funnel plot for the studies evaluating AT1R-Abs and its association with AR risk did not show asymmetry (see Figure 3). The Egger test (*p* = 0.47) and Begg test (*p* = 0.15) revealed no evidence of publication bias.

### 3.3. The Presence of AT1R-Abs and Risk of Kidney Graft Failure

**Figure 4:**
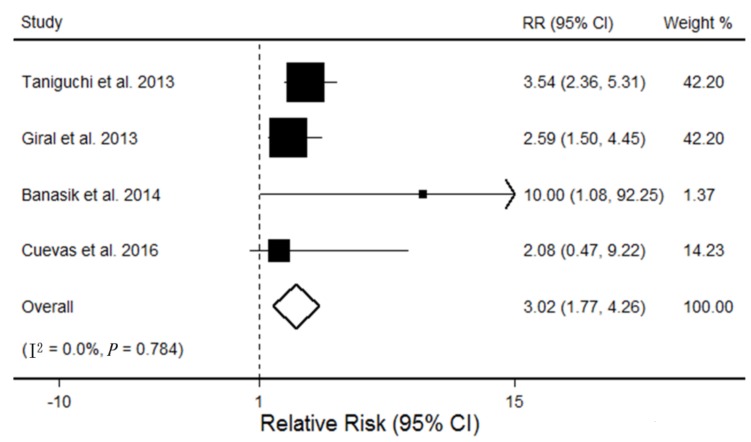
Forest plot on the association between AT1R-Abs and kidney graft failure. For each study, the estimation of RR and its 95% CI are plotted with a box and a horizontal line. The pooled odds ratio is represented by a diamond. The area of the gray squares reflects the weight of the study in the meta-analysis.

The association between AT1R-Abs and kidney graft failure was investigated in four studies comprising a total of 1208 participants [14,16,21,22]. Studies showed no existence of significant heterogeneity (I^2^ = 0.00%; *p* = 0.78), and the random-effects pooled incidence of kidney graft failure was significantly higher among patients with AT1R antibodies than those without AT1R antibodies (pooled RR, 3.02; 95% CI, 1.77–4.26; see Figure 4). We analyzed the effect of the difference in the endpoint of kidney graft failure between studies on the results in subgroup analysis. Inconsistencies in the endpoint did not alter the shape of the association (*p* = 0.55). Moreover, we conducted stratified analyses to evaluate whether the association of the presence of AT1R-Abs differs significantly between AR and kidney graft failure. The association of AT1R antibodies seemed to be slightly stronger with kidney graft failure than AR (*p* = 0.08), although statistical significance was not reached.

## 4. Discussion

In the present study, we evaluated the effects of AT1R-Abs on renal allograft outcome based on data from included studies. The results showed that AT1R-Abs were associated with an increased risk of AR and kidney graft failure. In addition, a significant correlation was found between a decrease in graft survival and the presence of AT1R-Abs, indicating that pretransplant detection of AT1R-Abs may be useful for identifying immunologic risks and kidney allograft outcome. Some stratified analyses across participant characteristics were conducted, with essentially no change in the results of the present study.

AT1R, a seven transmembrane-spanning G-protein-coupled receptor, is distributed in many cell types, including vascular endothelial cells and smooth muscle cells, and mediates physiological activities of angiotensin II under normal circumstances [25]; however, excessive activation of AT1R can trigger transcription factor expression in endothelial and vascular smooth muscle cells, which further results in the secretion of proinflammatory messengers. These inflammatory factors contribute to the pathological progression of vascular and renal diseases [26]. AT1R, as a vascular endothelium antigen, can be invariably recognized and combined by AT1R-Abs, which are produced by the host immune system after renal allograft transplant. As allosteric activators, AT1R-Abs could motivate AT1R in a similar manner as angiotensin II and further result in renal allograft injury [27,28]. The study conducted by Dragun et al. showed that the passive transfer of AT1R-Abs isolated from affected renal transplant patients into low-responder allogeneic rat transplant induced similar vascular rejection phenotypes as observed in their transplant biopsies [29]. In addition, the removal of AT1R-Abs by plasmapheresis in combination with AT1R blockade by Losartan has been proven to improve renal function and graft survival [30]. The results of our meta-analysis suggested that AT1R-Abs were significantly associated with increased risk of AR. The mechanism for such an observed relationship might relate to inflammation and coagulation induced by AT1R-Abs [31,32]. AT1R-Abs can regulate phosphorylation of extracellular signal-regulated kinase (ERK) 1/2, and further activate various nuclear transcription factors, including nuclear factor-kappa B (NF-κB) and the activator protein-1 (AP-1) in endothelial and vascular smooth muscle cells [33]. As important nuclear transcription factors, NF-κB can regulate several genes, including cytokines, adhesion molecules, and angiotensinogen involved in the pathogenesis of vascular inflammatory and acute rejection [34]. Furthermore, expression of tissue factor as regulated by NF-κB and AP-1 may increase procoagulatory activity of the injured vessels. Adhesion and chemotaxis of lymphocytes in the vascular bed of the allograft in response to vascular clotting may trigger acute rejection [35].

The present study showed that AT1R-Abs were also correlated with a significantly increased risk of kidney graft failure. The reasons for this might be that chronic rejection arises when low titer AT1R-Abs deposit on capillary endothelial surfaces and could activate inflammation and coagulation, which correlated with chronic allograft injury and transplant failure [11]. Moreover, this effect could be partly explained by the higher incidence of AR in patients with AT1R-Abs. Previous studies had suggested that AR was frequently accompanied by vascular or antibody-mediated rejection, which could initiate persistent chronic rejection followed by progressive functional decline to end-stage failure [36,37].

Our findings have important clinical health significance. Transplant rejection is closely associated with early and late graft loss. The prevalence of rejection episodes related to AT1R-Abs is thought to be 3.6% [29]. The present meta-analysis showed that the AT1R-Abs were significantly correlated with an elevated risk of AR and kidney graft failure, indicating that AT1R-Abs may be a potential biomarker for identification of patients at risk of AR and vascular injury that would be ignored by normal criteria. The assessment of pretransplantation AT1R-Abs status in patients could more effectively stratify the immunologic risk and optimize treatment strategy.

Several limitations of this study need to be considered. First, the present study was based on observational studies, which might be inherently biased by various factors. Second, although several confounding factors had been adjusted for in the included studies, the possibility of other uncontrolled or potential confounding factors could not be completely excluded in the present meta-analysis. Previous studies have shown that genetic or microenvironmental factors might also influence the severity and velocity of AT1R-Abs-associated pathologies, but we could not evaluate the effect of these factors due to insufficient data in the studies included [38]. Fourth, the relatively small sample sizes might influence the statistical power to assess the relationship. However, with negligible heterogeneity across studies, the results from the present meta-analysis were reliable. Fifth, although subgroup analysis showed that the difference in the endpoint of kidney graft failure between studies did not alter the shape of the association, the effect of the endpoint on the results needed further validation because of the small number of articles. Finally, although no publication bias was examined in the present study, it was still difficult to completely rule out such bias because there was not a sufficient number of studies to detect it adequately.

## 5. Conclusions

In conclusion, the present meta-analysis demonstrated that the AT1R-Abs were significantly associated with elevated risk of AR and kidney graft failure. Meanwhile, considering that only a few studies have examined this relationship, further well-designed, larger studies with prospective cohort design are required to validate the correlations between AT1R-Abs and renal graft outcomes.

## Figures and Tables

**Table 1:** Characteristics of studies included in this meta-analysis of anti-angiotensin type 1 receptor antibodies (AT1R-Abs) and kidney allograft outcomes.

	Study Participants (*n*)	Study Design	Sex (% Male)	Age (Years)	First Transplant	Patients with Living Donors	Detection of AT1R-Abs	Follow-Up Period	Diagnosis of AR	Classification of AR	Induction	Maintenance	Adjustment	Quality Score *
Robert et al., 2016 [23]	145 men and women in Australia (monocentric)	Cohort (HR)	66.2	51.3	87.6%	16.6%	ELISA	150 days	Allograft biopsies	Banff 2013	Anti-IL2R antibody 93.79%, ATG 6.21%	NR	NR	High
Philogene et al., 2016 [24]	70 men and women in US (monocentric)	Case- control (OR)	65.7	44.9	45.7%	75.7%	ELISA	NA	Allograft biopsies	Banff 2009–2013	91.43% Anti-IL2R antibody/ATG	TAC + MMF	NR	High
Cuevas et al., 2016 [16]	141 men and women in Mexico (monocentric)	Cohort (HR)	58.9	31.7	95.7%	NR	ELISA	3.5 years	Allograft biopsies	Banff 2007	Anti-IL2R antibody 75.10%, ATG 10.63%	MMF 87.9%	Donor age, Male-to-male donation, Class I %PRA	High
Lee et al., 2015 [12]	166 men and women in Korea (multicentric)	Cohort (HR)	66.9	45.7	95.2%	67.5%	ELISA	12 months	Allograft biopsies	Banff	Anti-IL2R antibody 96.39%, ATG 3.61%	TAC + MMF ± Steroid 76.51%, CsA + MMF + Steroid 13.25%, Others 10.24%	Gender, Age, mismatch ≥5, Peak PRA Class I > 0%, Peak PRA Class II > 0%, Pretransplant DSA, ABO incompatibility	High
In et al., 2014 [15]	79 men and women in Korea (monocentric)	Case-control (OR)	50.6	48.2	97.5%	65.8%	ELISA	NA	Allograft biopsies	Banff 2007	NR	NR	NR	High
Banasik et al., 2014 [14]	117 men and women in Poland (monocentric)	Cohort (HR)	66.7	47.7	94.0%	NR	ELISA	12 months	Allograft biopsies	Banff 2009	NR	TAC + MMF + Steroid 69.23%, CsA + MMF + Steroid 30.77%	Retransplantation, Historical peak PRA, HLA mismatch ≥ 5	High
Hernández-Méndez et al., 2014 [13]	103 men and women in Mexico (monocentric)	Cohort (RR)	54.4	27.7	97.1%	NR	ELISA	12 months	≥25% increase in serum creatinine	NR	Anti-IL2R antibody 80.58%, ATG 5.83%, None 12.62%	TAC + MMF + Steroid	de novo DSA, recipient age, donor age	High
Taniguchi et al., 2013 [22]	351 men and women in US (monocentric)	Case-control (OR)	56.1	48.3	91.7%	46.4%	ELISA	NA	Allograft biopsies	Banff 1997	Anti-IL2R antibody 41.6%, ATG 56.13%, Both 1.99%	TAC + MMF + Steroid 34.76%, CsA + MMF + Steroid 48.15%, Others 17.09%	Age, gender, race, primary disease, deceased donor, retransplant, pretransplant PRA > 10%, DGF, HLA mismatch, immunosuppression	High
Giral et al., 2013 [21]	599 men and women in French (monocentric)	Cohort (HR)	60.9	48.9	87.0%	94.2%	ELISA	4 months ^a^, 3 years ^b^	Allograftbiopsies	Banff 2007	Anti-IL2R antibody 49.6%, ATG 34%	TAC + MMF + Steroid 49.4%, CsA + MMF + Steroid 43.2%, Others 7.4%	HLA mismatch ≥ 5, Historical peak of anti-Class II PRA > 0%, Historical peak of anti-Class I PRA > 0%, Retransplantation	High

NR, not reported; AR, acute rejection; HRs, hazard ratios; OR, odds ratio; RR, relative risk; ELISA, enzyme linked immunosorbent assay; anti-IL2R, anti-human interleukin-2 receptor; DSA, donor-specific anti-HLA antibody; ATG, anti-thymocyte globulin; TAC, tacrolimus; CsA, cyclosporine A; MMF, mycophenolate mofetil; DGF, delayed graft function; PRA, panel reactive antibodies; HLA, human leukocyte antigen; ABO, ABO blood group system. ^a^ endpoint for AR; ^b^ endpoint for kidney graft failure; * study quality was judged based on Newcastle–Ottawa Scale.

**Table 2:** Stratified analysis of the association between AT1R-Abs and AR risk.

Subgroup	N	Pooled RR (95% CI)	*p*	
			Heterogeneity	Meta-Regression
Mean age (years)				
<46	4	1.47 (0.98, 1.95)	0.19	0.34
>46	5	1.95 (1.15, 2.74)	0.26	
Study type				
Cohort (RR)	1	2.47 (0.14, 4.80)	NA	0.11
Cohort (HR)	5	1.48 (0.88, 2.09)	0.34	
Case-control (OR)	3	1.77 (1.15, 2.39)	0.59	
Patients with living donors (%)				
>50	4	1.73 (0.71, 2.76)	0.59	0.56
<50	2	1.99 (1.26, 2.71)	0.69	
First transplant (%)				
>90	6	1.73 (1.28, 2.19)	0.06	0.33
<90	3	1.41 (0.29, 2.53)	0.89	
Adjustment				
Yes	3	1.66 (1.25, 2.07)	0.09	0.12
No	6	1.67 (0.04, 3.38)	0.71	
Country of origin				
America	5	1.55 (1.16, 1.93)	0.15	0.38
Europe	2	1.64 (0.37, 3.65)	0.58	

NA, not applicable because only one study.
